# Prevalence and risk factors of deep venous thrombosis in patients with longitudinally extensive transverse myelitis: one center data from China

**DOI:** 10.1186/s12883-018-1178-1

**Published:** 2018-10-30

**Authors:** Tian Song, Xindi Li, Yonghong Liu, Xinghu Zhang

**Affiliations:** 1 Neuroinfection and Neuroimmunology Center, Department of Neurology, Beijing Tiantan Hospital, Capital Medical University, 6 TiantanXili, Dongcheng District, Beijing, 100050 People’s Republic of China; 2 China National Clinical Research Center for Neurological Diseases, Beijing Tiantan Hospital, Capital Medical University, 6 TiantanXili, Dongcheng District, Beijing, 100050 People’s Republic of China; 3 Department of Neurology, Beijing Rehabilitation Hospital, Capital Medical University,Xixiazhuang, Shijingshan District, Beijing, 100050 People’s Republic of China

**Keywords:** Deep venous thrombosis, Longitudinally extensive transverse myelitis, Clinical risk factors, Thrombosis prophylaxis

## Abstract

**Objective:**

Deep venous thrombosis (DVT) is a severe complication in longitudinally extensive transverse myelitis (LETM) patients. It may interfere with LETM treatment and delay the recovery of the spinal dysfunction. However, there is less data about the prevalence and risk factors of DVT in patients with LETM. We analyzed data retrospectively to ascertain the prevalence of DVT and the clinical risk factors for DVT.

**Methods:**

Clinical data on 255 LETM patients were collected from medical records. All patients were performed color Doppler ultrasound(US) to screen DVT in both lower extremities when admitted. Clinical characteristics of LETM patients with DVT were compared with those without DVT using corresponding statistical methods. Multivariate logistic regression was performed to identify risk factors related to DVT.

**Results:**

DVT were found in 11.8% patients with LETM. Univariate analysis showed that age, muscle force and elevated baseline D-dimer were risk factors for DVT. After multivariate logistic regression, age, dyslipidemia, segments of lesions, and elevated baseline D-dimer remained significant independent risk factors.

**Conclusions:**

DVT is common in patients with LETM and related to patient’s age, dyslipidemia, segments of lesions, and elevated baseline D-dimer. Early recognition of DVT and thrombosis prophylaxis are appropriate in patients with LETM.

## Background

Longitudinally extensive transverse myelitis (LETM) is defined as myelitis with lesion extending at least 3 continuous vertebral segments in length [1]. A number of conditions can be associated with LETM, including neuromyelitis optica spectrum disorder (NMOSD), spinal infarction, spinal dural arteriovenous fistulas, compressive lesions, metabolic disorders, neoplasm, infection [2]. NMOSD is the most frequent cause of LETM [2], which primarily attacks the optic nerves and spinal cord [3]. LETM causes complete or partial neurologic impairment of motor, sensory and autonomic system [2]. These conditions are associated with increased morbidity and mortality, and a high economic burden [4]. Venous thromboembolism (VTE) is known as one of the common complications of LETM [5].

VTE includes deep venous thrombosis (DVT) and pulmonary embolism (PE). VTE affects 1/1000 patients, costs $13.5 billion annually to treat, and claims 100,000 lives annually in the US [6]. The risk of VTE in individuals with LETM may has been underestimated despite several plausible mechanisms. Autoimmune disorder associated with LETM may upregulate procoagulants, downregulate anticoagulants and suppress fibrinolysis [7].

But so far, little is known about the risk factors for DVT seen in LETM patients as it relates to other conditions, such as postoperation [8, 9], cancer [10, 11] and connective tissue diseases [12, 13]. Therefore, we evaluated the prevalence and risk factors of DVT and PE among patients with LETM in this study.

## Methods

We retrospectively collected the medical records of LETM inpatients from July 2011 to August 2014. All patients were performed color Doppler US to screen DVT in both lower extremities when admitted, regardless of symptoms and signs of DVT. US examinations were operated and interpreted by ultrasonic specialists. The deep veins of lower extremities and pelvis were screened at approximately 2-cm intervals in the transverse sections from the inguinal ligament to the ankle. A diagnosis of DVT was confirmed with the presence of visible embolus, dilated incompressible vessel, or abnormal flow pattern [14]. Similarly, D-dimer was tested in all patients when admitted, regardless of symptoms and signs of DVT. When patients complained dyspnea, tachypnea, or chest pain which could not be explained by other reasons, we tested PE using Computed Tomography Pulmonary Angiography (CTPA). Once VTE was detected, anticoagulant was administered.

Continuous variables were described by means±SDs, and categorical variables were described as percentages. Characteristics of patients with DVT were compared with those without DVT using corresponding statistical methods. Mann–Whitney U-test was performed for numerical variables (eg, patient’s age, inpatient days, inpatient costs, etiology of LETM, length of lesion). Fisher exact probability test was applied for the association of DVT and qualitative variables (eg, sex, combined diseases, location of lesions, contrast-enhanced or not, elevated baseline D-dimer). Ridit analysis was performed for ordinal variables, such as muscle strength of lower limbs. Logistic regression analysis was performed to identify patient characteristics related to DVT. Statistical significance was defined as *P* < 0.05. All statistical analysis was performed by IBM SPSS statistical software version 21.0 (IBM, Armonk, NY, USA).

The study protocol was approved by the ethics committee of Beijing Tiantan Hospital. Informed consent was signed by all patients or their legal representatives.

## Results

There were 255 LETM patients enrolled in this study in total. The diagnostic criteria for NMOSD were met in 116 patients (45.5%). In other 139 patients, an alternative specific diagnosis was made: infectious, 57; clinically isolated syndrome (CIS), 47; vascular myelopathy, 9; neoplastic, 5; compressive, 3; nutritional, 3; neurosarcoidosis, 2; and idiopathic, 13. 68 NMOSD cases were at first attack, and 48 cases were recurrent. These patients (186 women, 69 men) ranged in age from 15 to 77 years (mean 41.3 years). DVT was identified in 30 patients, corresponding to 11.8% of LETM cases, and 4 cases were symptomatic DVT. Symptomatic PE was confirmed in 1 patient by CTPA.

Patients with DVT were at older age, had poorer muscle force of lower limbs and higher baseline D-dimer when compared to those without DVT (Table 1, *P* < 0.001). The associations of lipid disorder, smoking and lesion length with DVT were nearly significant (*P* = 0.06) in univariate analysis. The duration of hospitalization was obviously longer in patients with DVT than that without DVT (31.87 versus 18.95 days, *P* < 0.001). The hospital charges were higher in patients with DVT than patients absence of DVT (38,178 versus 17,153 CNY, *P* < 0.001). There were no differences in sex, comorbidity, etiology, location and enhancement of lesions, and coagulogram abnormal.

**Table 1:** Characteristics of LETM patients with and without DVT

Variable	Total (*N* = 255)	With DVT (*N* = 30)	Without DVT (*N* = 225)	*P* value
Age (years), mean ± SD	41.3 ± 13.93	53.73 ± 11.94	39.65 ± 13.34	< 0.001
Sex, *n*(%)				
Men	69	7(23.33%)	62(27.56%)	0.62
Women	186	23(76.67%)	163(72.44%)	
Inpatient stays (days), mean ± SD	20.47 ± 9.90	31.87 ± 17.68	18.95 ± 7.14	< 0.001
Inpatient costs (CNY), mean ± SD	19,627.22±14,019.56	38,178.44 ± 30,309.69	17,153.73 ± 7190.18	< 0.001
Comorbidity, *n*(%)				
Hematological abnormality (anemia, leukopenia)	13(5.10%)	1(3.33%)	12(5.33%)	0.64
Connective tissue diseases	39(15.29%)	5(16.67%)	34(15.11%)	0.82
Thyroid dysfunction	22(8.63%)	3(10.00%)	19(8.44%)	0.78
Diabetes mellitus type 2	12(4.71%)	3(10.00%)	9(4.00%)	0.14
Hypertension	27(10.59%)	5(16.67%)	22(9.78%)	0.25
Dyslipidemia	80(31.37%)	5(16.67%)	75(33.33%)	0.06
Smoking	42(16.47%)	9(30%)	33(14.67%)	0.06
Etiology, *n*(%)				
NMOSD	116(45.49%)	13(43.33%)	103(45.78%)	0.85
First attack	68(26.67%)	6(20%)	62(27.56%)	0.38
Relapses	48(18.82%)	7(23.33%)	41(18.22%)	
Infection	57(22.35%)	9(30%)	48(21.33%)	0.35
CIS	47(18.43%)	4(13.33%)	43(19.11%)	0.62
Vascular myelopathy	9(3.53%)	1(3.33%)	8(3.56%)	0.71
Neoplastic	5(1.96%)	1(3.33%)	4(1.78%)	0.47
Compressive	3(1.18%)	0	3(1.33%)	1.00
Nutritional	3(1.18%)	0	3(1.33%)	1.00
Neurosarcoidosis	2(0.78%)	0	2(0.89%)	1.00
Idiopathic	13(5.10%)	2(6.67%)	11(4.89%)	0.66
Muscle strength of left leg, *n* (%)				
Grade 0	19(7.45%)	8(26.67%)	11(4.89%)	< 0.001
Grade 1	7(2.75%)	2(6.67%)	5(2.22%)	
Grade 2	14(5.49%)	5(16.67%)	9(4.00%)	
Grade 3	25(9.80%)	4(13.33%)	21(9.33%)	
Grade 4	73(28.63%)	5(16.67%)	68(30.22%)	
Grade 5	117(45.88%)	6(20.00%)	111(49.33%)	
Muscle strength of right leg, *n* (%)				
Grade 0	19(7.45%)	8(26.67%)	11(4.89%)	< 0.001
Grade 1	10(3.92%)	4(13.33%)	6(2.67%)	
Grade 2	11(4.31%)	4(13.33%)	7(3.11%)	
Grade 3	21(8.24%)	4(13.33%)	17(7.56%)	
Grade 4	68(26.67%)	8(26.67%)	60(26.67%)	
Grade 5	126(49.41%)	2(6.67%)	124(55.11%)	
Location of lesions, *n* (%)				
Cervical	204(80.00%)	25(83.33%)	179(79.56%)	0.63
Thoracic	133(52.16%)	17(56.67%)	116(51.56%)	0.60
Lumbar	8(3.14%)	1(3.33%)	7(3.11%)	1.00
Segments of lesions, mean ± SD	5.20 ± 3.56	6.77 ± 4.98	5 ± 3.30	0.06
Enhancement of lesions, *n* (%)				
Yes	115(45.10%)	13(43.33%)	102(45.33%)	0.60
No	108(42.35%)	11(36.67%)	97(43.11%)	
Unknown	32(12.55%)	6(20.00%)	26(11.56%)	
Internals between LETM symptoms onset and US screening(days), mean ± SD	18.45 ± 7.40	21.40 ± 10.26	18.07 ± 6.87	0.025
Coagulogram abnormal, n (%)	108(42.35%)	14(46.67%)	94(41.78%)	0.61
Baseline D-dimer elevated, n (%)	24(9.41%)	17(56.67%)	7(3.11%)	<0.001

Multivariate logistic regression analysis presented that elderly patients showed higher likelihood of DVT with an OR of 1.10(95% CI 1.03–1.18). In addition, lipid disorder (OR0.07, 95%CI 0.01–0.51), lesion segments (OR1.53, 95%CI 1.14–2.05), elevated baseline D-dimer (OR18.40, 95%CI 3.32–101.89) were independent risk factors (*P* < 0.05) for occurrence of DVT (Table 2).

**Table 2:** Multivariate logistic regression analysis of significant risk factors for DVT in LETM patients

	*P*	OR(95% CI)
Age	0.004	1.104(1.032–1.181)
Dyslipidemia	0.009	0.07(0.01–0.51)
Segments of lesions	0.005	1.529(1.140–2.050)
Baseline D-dimer elevated	< 0.001	18.395(3.321–101.891)

## Discussion

This is a retrospective and observational study to identify the prevalence and risk factors of DVT in LETM patients. DVT prevalence among LETM inpatients in our study was 11.8%, obviously higher than that in non-surgical inpatients [11, 15], outpatients [16] and community population [17]. However, previous studies regarding to surgical and spinal cord lesion cases showed inconsistent DVT prevalence, ranging from 1.2% [18] to 27.6% [19]. This inconsistence can be explained by difference in population/race, sample size, DVT definition (symptomatic or asymptomatic).

Another primary aim of this study was to analyzed individual-level risk factors of DVT. Our data showed that age, dyslipidemia, lesion segments and elevated baseline D-dimer remained independent risk factors after adjustment for covariates. There are numerous published reports concerning the risk factors for DVT development in various diseases. A study about patients after spine surgery reported that risk factors for DVT included age and preoperative walking disability [20]. Immobility as a risk factor of DVT was also mentioned in neurosurgical patients, including cranial and spinal procedures [9]. However, there were different opinions. A Korean-based study reported that age and completeness of motor paralysis were not significantly associated with occurrence of DVT after spinal cord injury [21]. In our study, immobility appeared to be a risk factor in univariate analysis, but it was no longer independent risk factor after multivariate analysis. D-dimer values, in particular age-adjusted D-dimer cut-off values, have been confirmed to be associated with DVT [22, 23]. Our finding is in accordance with above researches. A review showed lipoprotein (a) was slightly but significantly associated with an increased risk of VTE (OR: 1.56, 95% CI: 1.36, 1.79), which included 10 studies, 13,541 patients [24]. Our study analyzed dyslipidemia as a whole including hyper/hypo-triglyceridemia and hyper/hypo- hypercholesteremia, instead of lipoprotein (a) individually. Much of the differences between studies may be due to small patient populations and different methods for assessment of DVT [25]. We have not searched literature focused on risk factors of DVT in LETM patients. Therefore,larger prospective studies will be needed to illuminate the risk factors for DVT and intrinsic mechanism.

The plausible mechanisms to explain the high prevalence of DVT in LETM patients remain unclear. Three main promoters to venous thrombosis included: venous stasis, hypercoagulable blood state and vascular endothelial injury (Virchow’s triad) [26]. Immobility caused by spinal cord dysfunction can lead to venous stasis in LETM cases. There is some evidence that steroid use can increase procoagulant factors [27]. Most NMOSD and CIS patients in our study received high dose steroid pulse therapy. In addition, hospitalization also is a known risk factor for VTE [28]. Above elements may contribute to the high prevalence of DVT in LETM inpatients.

Several limitations to our analysis are worth noting. First limitation is lack of prospective control, which is inherent to observational studies. Second, the interval was not standardized between LETM symptom onset and US screening. The intervals in patients with DVT were significantly longer (*P* < 0.05, Table 1). The prevalence in our study might be underestimated because US screening was performed beyond the acute phase in some cases. Third, certain confounders were unavailable in our databases, such as body mass index(BMI) [29] and glucocorticoid usage [30], which are known as risk factors for DVT. Despite these limitations, our data is convincing for the large sample size and routine US screening for DVT.

## Conclusion

In conclusion, this retrospective study demonstrated that patients with LETM had an increased risk for developing DVT. Therefore, it is important to identify the high-risk patients and initiate thrombosis prophylaxis promptly.

## Abbreviations

BMI: body mass index
CIS: Clinical isolated syndrome
CNS: Central nervous system
CTPA: Computed Tomography Pulmonary Angiography
DVT: Deep venous thrombosis
LETM: Longitudinally extensive transverse myelitis
NMOSD: Neuromyelitis optica spectrum disorders
PE: Pulmonary embolism
US: Ultrasound
VTE: Venous thromboembolism
